# A rare extrapulmonary presentation of tuberculosis: Breast tuberculosis

**DOI:** 10.1002/ccr3.7728

**Published:** 2023-07-23

**Authors:** Shambhu Khanal, Anil Kafle, Sushmita Bhandari, Priyanka Kayastha

**Affiliations:** ^1^ Department of Internal Medicine Tribhuvan University Institute of Medicine Kathmandu Nepal; ^2^ Department of Pathology BP Koirala Institute of Health Sciences Dharan Nepal; ^3^ Department of Surgery Fatima Jinnah Medical University Lahore Pakistan; ^4^ Department of Pathology National Academy of Medical Sciences Kathmandu Nepal

**Keywords:** breast lump, breast tuberculosis, fine needle aspiration cytology, ZN stain

## Abstract

**Key Clinical Message:**

Breast abscess of long duration can be tubercular origin in both developing and developed countries despite its rarity.

**Abstract:**

A 34‐year‐old lady presented with painful lump on her breast for 2 months, which was diagnosed with mammary tuberculosis on basis of aspiration cytology and successfully treated with antitubercular drugs. Breast abscess of long duration may be tubercular etiology in both developing and developed nations.

## 1 INTRODUCTION

Breast tuberculosis (TB) is a rare extra pulmonary occurrence even in developing countries where the prevalence of TB is high with an incidence of 3.0%–4.5% in surgically treated breast diseases.^1^ The overall incidence of breast TB is less than 0.1% of breast diseases.^1^ Among the variable clinical presentation of mammary TB, solitary lump and enlarged axillary lymph nodes are the commonest one.^2^ The diagnosis of the breast tuberculosis poses a great challenge to clinicians because the features mimic with malignancy as well abscess and TB can easily be overlooked considering its site for presentation. Standard diagnosis is based on Ziehl–Neelsen (ZN) staining and culture for the confirmation, which is not readily available everywhere. However, the breast TB should be a differential in breast lumps in developing countries where prevalence of TB is high. Hereby, we present a case of 34‐year‐old female with lump in breast, which was later diagnosed as mammary TB on fine needle aspiration cytology (FNAC).

## 2 CASE REPORT

A 34‐year‐old lady from countryside presented to the outpatient department of our hospital with painful lump on her right breast since 2 months. She had no history of fever, cough shortness of breath, weight loss, anorexia, and trauma. She was treated as pyogenic abscess with oral antibiotics from the local health center. She had no history of known exposure to microbiologically confirmed tuberculosis case. On examination, the lump was painful, firm, regular, fluctuant, and non‐adherent to overlying skin measuring 5 cm by 3 cm. She had axillary lymphadenopathy with a mobile firm 2 cm by 2 cm lymph node on right axilla.

Her hemogram report showed hemoglobin 10.3 g/dL, white blood cell count 8000/cumm, neutrophil 66%, lymphocyte 28%, eosinophil 6%. Her renal function was within normal range with urea 20 mg/dL, serum creatinine 0.8 mg/dL, sodium 138 meq/L, potassium 4 meq/L. Her liver function tests were also normal. The chest x‐ray was noncontributory. Pus Gram stain and culture showed no organism.

Ultrasound of the right breast showed heterogenous hypoechoic lesion with internal echoes and no internal vascularity suggestive of breast abscess.

**FIGURE 1:**
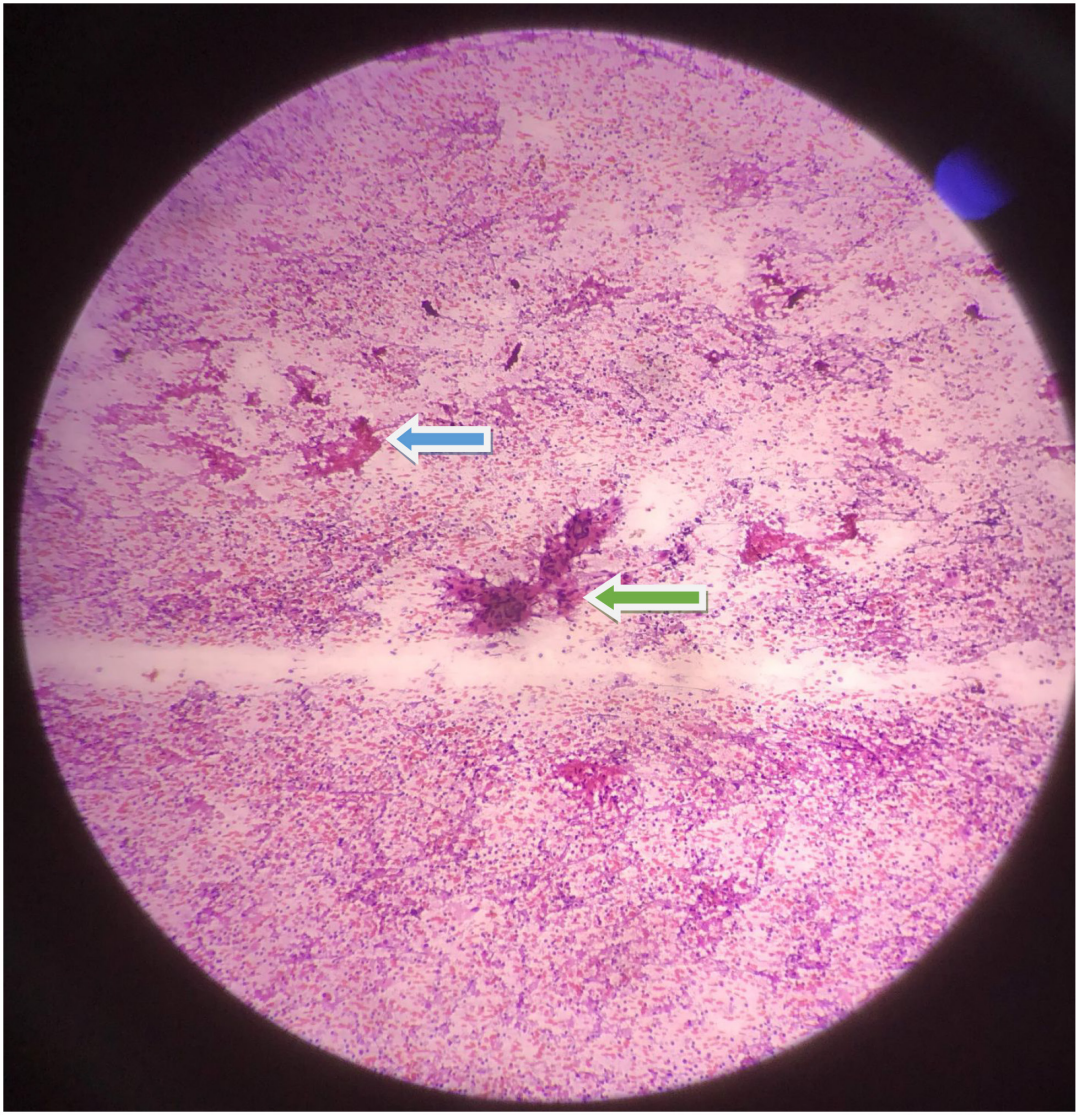
FNA cytology smear of axillary lymph node shows epitheloid granuloma(green arrow), necrosis (blue arrow), and lymphocytes (Papanicolaou stain).

**FIGURE 2:**
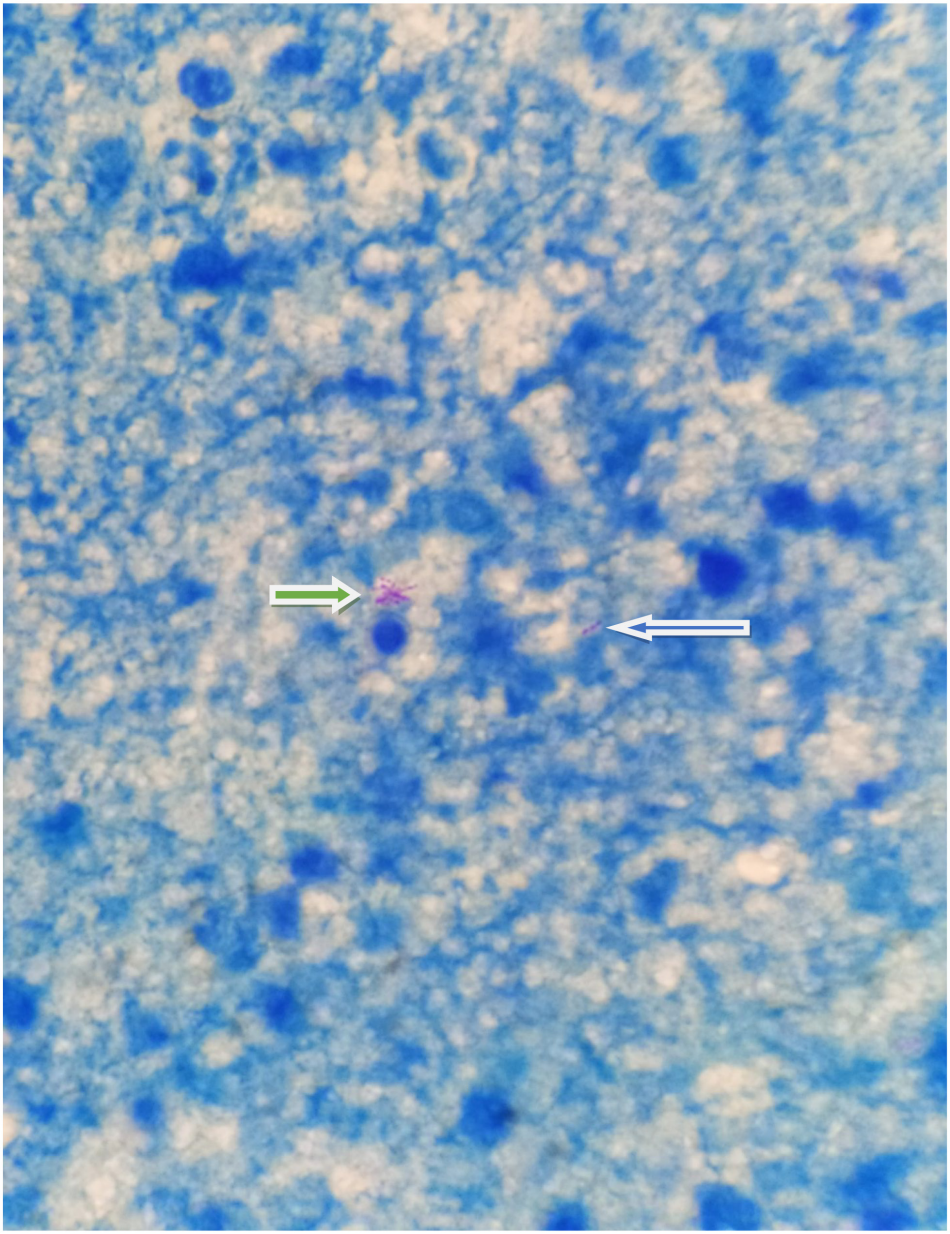
Smear shows single acid fast bacilli (blue arrow) and aggregates of acid fast bacilli (green arrow) having rod shaped beaded and eosinophilic morphology in Ziehl–Neelsen stain.

On fine needle aspiration cytology from the axillary lymph node, it showed mature transforming lymphocytes, lymphohistiocytic aggregates, some with epitheloid morphology forming granuloma and granulation tissue fragments against dirty background as shown in Figure 1. On aspirate from lump, it revealed predominant necrosis along with occasional lymphocytes and histiocytes. The ZN staining from the aspirate of lymph node showed acid fast bacilli as shown in Figure 2. Owing to the unavailability of culture method and paucity of gene xpert, the aspirate could not be sent for mycobacterium gene xpert and culture.

She was started on antitubercular drug combination comprising isoniazid, rifampicin, pyrazinamide and ethambutol. The pain and size of swelling subsided gradually. She completed her total 6 months course of treatment as per national guidelines. Intensive phase included isoniazid, rifampicin, pyrazinamide and ethambutol for 2 months and then continuation phase included isoniazid and rifampicin for 4 months. The lump in the breast subsided completely without need of surgical intervention.

## 3 DISCUSSION

Primary tuberculosis in breast is an uncommon presentation of tuberculosis in the areas of high prevalence as well. Like skeletal muscles and spleen, the breast tissue is resistant to infection and multiplication of mycobacterium tuberculosis.^3^ The foci of tuberculosis elsewhere in body remain quiescent for unknown reason and is quite rare in TB breast. However, the breast tuberculosis has been considered as secondary involvement by authors.^4^ The mean time for onset of symptoms and diagnosis of TB breast is 7.7 months (range 3–12 months) owing to the resemblance with other benign and malignant breast diseases as in our case with pyogenic breast abscess.^5^ Breastfeeding seems to be protective against breast cancer but predisposes to breast abscess owing to repeated microtrauma.^6^ Although both breasts are susceptible to infection, bilateral involvement is found in only 3% cases.^7^ The breast lump with or without ulceration is the most common clinical presentation of breast TB.^1^ Constitutional symptoms of TB like fever, weight loss, anorexia, malaise, and poor general health are rarely encountered in mammary TB.^1^ FNAC stands as an important method for diagnosis of breast TB. Some authors claim the sensitivity of FNAC in breast TB to be 73%.^(^ ^4^, ^8^ ^)^ About 5% cases of TB breast are diagnosed both by histopathological and ZN staining. The presence of chronic granulomatous inflammation and Langerhans's giant cell with caseous necrosis seems sufficient for diagnostic confirmation for TB breast in 95% cases.^5^ As in our case, cytopathological examination of aspirate from breast lump showed only necrosis. The FNAC from the axillary lymph node showed necrosis and ZN stain positive for acid fast bacilli as shown in Figure 1. Concomitant axillary lymph node with breast lump is found in one‐third of patient.^1^ Where there is high index of suspicion regarding TB breast, the anti‐tubercular dugs should be started as early to prevent fistula formation.^9^ The duration of treatment is at least 6 months with 2 months of intensive phase and 4 months of continuation phase as in pulmonary TB. The intensive phase comprises isoniazid (H), rifampicin(R), pyrazinamide and ethambutol(E). The continuation phase comprises of isoniazid and rifampicin. Surgical excision may be needed in cases with poor response to antitubercular drugs or large painful ulcerated lesions. Simple mastectomy may be rarely needed in case of large extensive involvement of breast and draining axillary lymph nodes.^1^, ^3^, ^6^

## 4 CONCLUSION

Tuberculosis may primarily involve breast without any constitutional symptoms and obvious evidence of tubercular foci elsewhere in body. Cytopathology study can help in correct diagnosis and treatment. Tuberculosis should be kept as a differential in case of lump in breast in both developing and developed nations despite its rarity of breast involvement.

## AUTHOR CONTRIBUTIONS

**Shambhu Khanal:** Conceptualization; formal analysis; writing – original draft. **Anil Kafle:** Resources; writing – original draft. **Sushmita Bhandari:** Resources; writing – review and editing. **Priyanka Kayastha:** Writing – review and editing.

## FUNDING INFORMATION

No funding source for this work.

## CONFLICT OF INTEREST STATEMENT

The authors declare that there is no conflict of interest regarding publication of this case report.

## ETHICS STATEMENT

Need for ethical approval was waived. Consent from the patient deemed to be enough.

## CONSENT

Written informed consent was taken from the patient for the publication of the case report. A copy of the consent form will be available for review if asked by editor in chief of journal.

## Data Availability

The data that support the findings of this study are available from the corresponding author upon reasonable request.
